# 919. Let’s GO PO: Impact of Monthly Feedback on a Longitudinal Intravenous to Oral Antimicrobial Conversion Initiative

**DOI:** 10.1093/ofid/ofac492.764

**Published:** 2022-12-15

**Authors:** Jillian E Hayes, Amy L Carr

**Affiliations:** Duke University Hospital, Durham, North Carolina; AdventHealth Orlando, Orlando, Florida

## Abstract

**Background:**

Timely conversion of antimicrobials from intravenous (IV) to oral (PO) route has been shown to decrease costs and length of stay (LOS) without compromising safety and efficacy of therapy. Use of oral antimicrobials may additionally prevent complications related to IV catheters, such as infection, emboli, and patient discomfort. An approved IV to PO policy allowed pharmacists to convert orders for fourteen included antimicrobials and eligible patients at time of order verification.

**Methods:**

This single-center, retrospective, comparative study was conducted at AdventHealth Orlando, a 1,368-bed community teaching hospital in central Florida. In November 2020, six clinical pharmacist teams began receiving monthly feedback on IV to PO conversion rates in the form of a RePOrt Card, containing IV to PO conversion rates compared to other clinical teams, individual antimicrobial conversion rates, and comparison to prior team progress (Figure 1). RePOrt Cards were provided through October 2021. This study compared pre-intervention days of therapy (DOTs) of antimicrobials from November 2019-October 2020 to post-intervention DOTs from November 2020 to March 2022. The primary objective of this study was to assess the impact of monthly, team-based feedback on percentage of antimicrobials administered orally during a pharmacist-driven IV to PO stewardship initiative.

**Figure 1:**
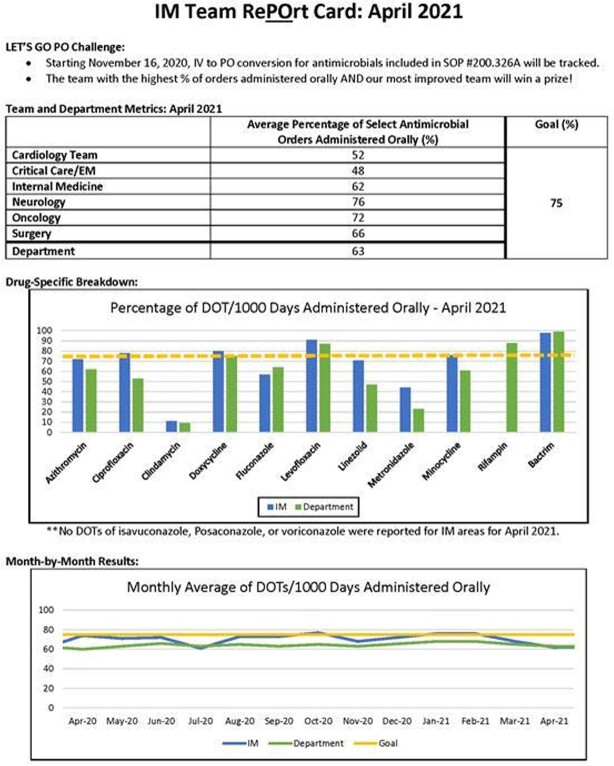
Example RePOrt Card

**Results:**

Significantly more DOTs were administered orally in the post intervention group (62% vs 67%, p=0.0012). Positive change in oral conversion rates was observed for all agents except linezolid, minocycline, and voriconazole (Table 1). The largest increase in percentage of DOT administered orally was observed for azithromycin (20%), rifampin (14%), and metronidazole (10%). Estimated monthly and total cost differences are available in Table 2. Minocycline represents the largest opportunity missed; while oral conversion rates remained the same, an increase in overall drug use creates opportunity to continue to prioritize oral conversion due to high cost.

**Figure:**
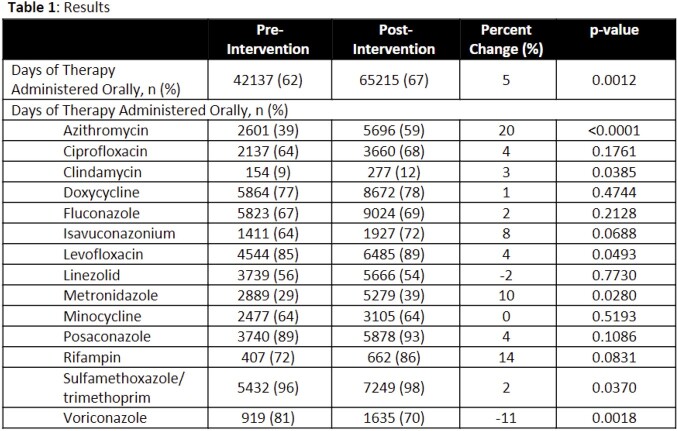

**Figure:**
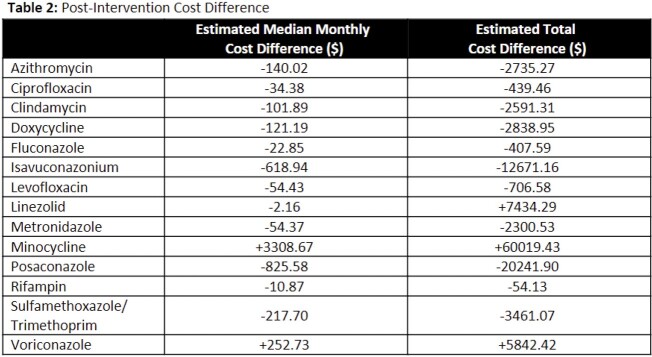

**Conclusion:**

Monthly, team-based feedback positively impacted IV to PO conversion rates. Opportunities remain to optimize cost benefits in high-cost agents such as linezolid, minocycline, and voriconazole.

**Disclosures:**

**Amy L. Carr, PharmD, BCIDP**, Shionogi: Advisory Board.

